# Evolution of Friedreich’s Ataxia Management Across Established and Emerging Therapies—Systematic Review and Meta-Analysis

**DOI:** 10.3390/jcm15145707

**Published:** 2026-07-21

**Authors:** Basel Garah, Hisham Aljabri, Abdullah Aljohani, Ethar Alnuzha, Arwa Maihoub, Reenad Almuzaini, Layan Aljohani, Layan Alshamani, Majed Alluqmani

**Affiliations:** College of Medicine, Taibah University, Madinah 42353, Saudi Arabiamloqmani@hotmail.com (M.A.)

**Keywords:** Friedreich ataxia, FDA, omaveloxolone, genetic, neurology

## Abstract

**Background:** Friedreich’s ataxia (FRDA) is a neurodegenerative disorder driven by frataxin deficiency, resulting in mitochondrial dysfunction and reduced nuclear factor erythroid 2-related factor 2 (Nrf2) signaling. Pharmacologic trials have yielded inconsistent results, prompting an updated synthesis of evidence. **Methods:** We searched MEDLINE/PubMed, Google Scholar, Cochrane CENTRAL, ClinicalTrials.gov, and the World Health Organization (WHO) International Clinical Trials Registry Platform (ICTRP) from database inception to 29 June 2025. Embase, Scopus and Web of Science were not searched due to institutional access limitations. Two reviewers independently screened studies, extracted data, and assessed risk of bias. Random-effects meta-analyses were conducted, and Grading of Recommendations, Assessment, Development, and Evaluations (GRADE) was used. Primary outcomes were modified Friedreich Ataxia Rating Scale (mFARS)/Friedreich Ataxia Rating Scale (FARS); safety outcomes included adverse events (AEs) and serious AEs. Secondary outcomes were Scale for the Assessment and Rating of Ataxia (SARA), International Cooperative Ataxia Rating Scale (ICARS), Nine-Hole Peg Test, and the Timed 25-Foot Walk. **Results:** Sixteen studies (17 reports, *n* = 351) met inclusion criteria. Omaveloxolone was the only agent showing a statistically significant improvement in mFARS (mean difference (MD) −2.40; 95% confidence interval (CI) −4.24 to −0.56; *p* = 0.014), supported by low-certainty evidence. Other therapies showed no consistent benefit. Overall AE risk was comparable to control (risk ratio (RR) 1.00; 95% CI 0.98–1.03). Apparent subgroup differences by therapeutic class or age likely reflected drug-specific effects and small samples. **Conclusions:** Omaveloxolone was the only agent to reach statistical significance for mFARS and is the most promising and best-supported therapy among those reviewed; however, this rests on low-certainty evidence and needs confirmation in larger trials. No clear difference in overall adverse events was observed between intervention and control groups; however, available safety evidence remains limited by imprecision, small sample sizes, and short follow-up durations. Longer, standardized, and age-stratified randomized controlled trials (RCTs) are needed.

## 1. Introduction

Friedreich’s ataxia (FRDA) is a rare, autosomal recessive neurodegenerative disorder caused by GAA trinucleotide repeat expansion in the FXN gene, which lowers production of frataxin—a mitochondrial protein essential for iron–sulfur cluster assembly and energy generation. Clinically, FRDA presents with progressive gait and limb ataxia, dysarthria, scoliosis, cardiomyopathy, metabolic disturbances and, in severe cases, respiratory failure. At the cellular level, frataxin deficiency produces mitochondrial dysfunction marked by oxidative stress, reduced adenosine triphosphate (ATP) generation and mitochondrial iron accumulation; recent studies also show downregulation of Nrf2 signaling, weakening antioxidant defenses and accelerating neuronal death [1,2].

Therapeutically, efforts initially targeted oxidative stress and iron overload using antioxidants and iron chelators, with promising preclinical results but limited and inconsistent clinical efficacy. The therapeutic landscape shifted with FDA approval of omaveloxolone, an Nrf2 activator that improves mitochondrial bioenergetics and antioxidant capacity and is the first pharmacologic therapy approved for FRDA. Other agents under study include idebenone, deferiprone, EPI-743 (vatiquinone), interferon-γ-1b and numerous experimental compounds, yet there is no consolidated, comparative review of their efficacy and safety. This systematic review and meta-analysis therefore aims to synthesize evidence on clinical efficacy, neurological outcomes, disease progression and safety across established, approved and emerging pharmacological treatments for FRDA to clarify current options and guide future research [1,2,3,4].

## 2. Rationale and Objectives

We aim to systematically evaluate and compare the efficacy and safety of pharmacological interventions for FRDA relative to placebo or standard care, by pooling and synthesizing evidence across available randomized controlled trials. Given the heterogeneity of pharmacological approaches currently under investigation for FRDA and the absence of an approved disease-modifying therapy, cross-trial comparison is essential to identify which interventions demonstrate the most consistent clinical benefit, inform future trial design, and provide an evidence-based overview of the current FRDA treatment landscape.

## 3. Materials and Methods

### 3.1. Protocol and Registration

This systematic review and meta-analysis followed the Preferred Reporting Items for Systematic Reviews and Meta-Analyses (PRISMA) 2020 guidelines and was prospectively registered on International Prospective Register of Systematic Reviews (PROSPERO)ATP (CRD420251076369) (Supplementary Table S1, Prisma 2020 checklist). The protocol was finalized before any project steps to minimize bias and ensure transparent, pre-specified methods covering study selection, data extraction, risk-of-bias assessment, and statistical analyses.

### 3.2. Eligibility Criteria

We included randomized controlled trials (RCTs), quasi-randomized trials, non-randomized comparative studies, and single-arm interventional studies evaluating pharmacologic therapies in patients with confirmed Friedreich’s ataxia. Eligible participants included individuals of any age with confirmed Friedreich’s ataxia. We included studies investigating any systemic pharmacologic intervention administered for therapeutic purposes, regardless of dose, formulation, or duration. Eligible comparators included placebo, no treatment, standard care, active comparators, or historical controls. For inclusion, studies were required to report at least one efficacy outcome measure (validated ataxia rating scales including the modified Friedreich Ataxia Rating Scale (mFARS), Friedreich Ataxia Rating Scale (FARS), Scale for Assessment and Rating of Ataxia (SARA), International Cooperative Ataxia Rating Scale (ICARS), or functional performance tests) or safety outcomes (adverse events (AEs), serious adverse events (SAEs), or treatment discontinuations).

We excluded studies focusing only on non-pharmacologic interventions such as physical therapy, surgical procedures, or medical devices. We also excluded case reports, case series with fewer than five participants, conference abstracts without full-text publications, preclinical studies, and review articles such as narrative reviews, systematic reviews, and meta-analyses. We included only studies with full-text available in English, excluding studies that did not fulfil this criterion.

### 3.3. Information Sources and Search Strategy

We conducted a literature search of multiple electronic sources, including PubMed, Google Scholar, Cochrane CENTRAL, ClinicalTrials.gov, and the WHO International Clinical Trials Registry Platform (ICTRP) from database inception to 29 June 2025. Embase was not searched due to institutional access limitations; MEDLINE was not searched separately because PubMed substantially overlaps with its content (see Limitations). In addition, reference lists of included studies and relevant review articles were hand-searched to identify additional eligible studies through backward citation tracking. The full search strings for each database and registry are provided in Supplementary Materials.

The search strategy was developed using a combination of Medical Subject Headings (MeSH) terms and free-text keywords. The search syntax and key terms included the following elements: (“Friedreich ataxia” OR “Friedreich’s ataxia” OR “FRDA” OR “spinocerebellar degeneration” OR “frataxin deficiency” OR ((frataxin OR FXN OR FRDA) AND (deficiency OR mutation OR “GAA repeat” OR “trinucleotide repeat”))) AND (“drug therapy” OR pharmacotherapy OR treatment OR therapy OR intervention OR “clinical trial” OR “randomized controlled trial” OR RCT OR “controlled clinical trial” OR trial OR drug OR medication OR pharmaceutical OR agent OR compound OR idebenone OR omaveloxolone OR deferiprone OR interferon OR “histone deacetylase inhibitor” OR HDAC OR resveratrol OR “coenzyme Q” OR CoQ10 OR antioxidant OR “neuroprotective agent” OR erythropoietin OR leriglitazone OR vatiquinone OR “iron chelation” OR “mitochondrial therapy” OR “Nrf2 activator”). The search strategy was properly adapted for each database using their respective controlled vocabularies and syntax requirements. The full search strings for CENTRAL, ClinicalTrials.gov, and WHO ICTRP are provided in Supplementary Materials.

### 3.4. Study Selection

Two reviewers independently screened studies, with any conflict resolved by a third reviewer. All records identified through the literature search were imported into reference management software, and duplicate records were removed using manual deduplication processes. We first conducted title and abstract screening of all unique records to identify potentially eligible studies. Full-text articles were then obtained for all records meeting initial screening criteria or where eligibility could not be determined from the title and abstract alone. We then assessed full-text articles against our predefined eligibility criteria.

### 3.5. Data Collection Process

Two reviewers independently extracted data, with any conflict resolved by a third reviewer. Extracted information included study characteristics (first author, publication year, study design, phase, registration number, country, funding source), participant demographics (sample size, age, gender distribution, disease duration, GAA repeat length, baseline disease severity), intervention details (drug name, dose, route of administration, treatment duration, comparison group), and outcome data (means, standard deviations, change scores, effect estimates, confidence intervals, *p*-values for all reported outcomes).

For studies with multiple treatment arms, we extracted data for all relevant comparisons. When studies reported outcomes at multiple time points, we extracted data from the primary endpoint as specified by the study authors or, if not specified, the longest follow-up duration.

### 3.6. Risk of Bias Assessment

Two reviewers independently assessed risk of bias, with any conflict resolved by a third reviewer. We assessed the risk of bias in included RCTs using the revised Cochrane Risk of Bias tool (RoB 2.0), which evaluates five domains: bias arising from the randomization process, bias due to deviations from intended interventions, bias due to missing outcome data, bias in measurement of the outcome, and bias in selection of the reported result. Each domain was rated as low risk, some concerns, or high risk of bias based on responses to signaling questions and domain-level algorithms. For non-randomized studies of interventions, we applied the Risk of Bias in Non-randomized Studies of Interventions (ROBINS-I) tool, assessing seven domains including confounding, selection of participants, classification of interventions, deviations from intended interventions, missing data, measurement of outcomes, and selection of reported results. Phase I safety studies and single-arm studies without comparators were not assessed using these comparative bias tools as they are not designed for such study designs.

### 3.7. Data Synthesis and Statistical Analysis

We conducted quantitative synthesis using random-effects meta-analysis models when two or more studies reported comparable outcome measures with sufficient data for pooling. Quantitative efficacy and safety syntheses were restricted to randomized controlled trials (RCTs). To avoid double-counting participants, the three reports of the omaveloxolone MOXIe program were treated as distinct units: Part 1 [5] and Part 2 [2] enrolled separate, non-overlapping cohorts and were included as independent comparisons, whereas the delayed-start extension [6] re-followed the Part 2 cohort and was therefore excluded from all pooled estimates and used in narrative synthesis only. Non-randomized, single-arm, and phase I studies were included in the systematic review but were summarized narratively. Studies were pooled only when they evaluated the same drug or mechanistic class; we did not combine different pharmacological classes into a single efficacy estimate, as such an estimate is not clinically interpretable. Where only one study reported an outcome, or where interventions were too heterogeneous to combine, findings were summarized narratively. For continuous outcomes, we calculated mean differences (MDs) with corresponding standard errors and 95% confidence intervals (CIs). When studies reported change-from-baseline scores without standard deviations, we imputed missing standard deviations using the Cochrane Handbook formula with an assumed correlation coefficient of 0.5 between baseline and follow-up measurements, with sensitivity analyses conducted varying this correlation coefficient from 0.4 to 0.7.

For dichotomous safety outcomes, we calculated risk ratios (RRs) with 95% CIs. Statistical heterogeneity was quantified using the I^2^ statistic and interpreted according to established thresholds (0–40% might not be important, 30–60% may represent moderate heterogeneity, 50–90% may represent high heterogeneity, 75–100% significant heterogeneity), with corresponding τ^2^ values reported to quantify between-study variance. The chi-square test for heterogeneity was also performed, with *p*-values less than 0.10 considered indicative of significant heterogeneity given the typically low power of this test. All meta-analyses were conducted using random-effects models based on the DerSimonian and Laird method to integrate and account for between-study heterogeneity, except for sensitivity analyses comparing fixed-effect model results.

### 3.8. Subgroup and Sensitivity Analyses

We performed subgroup analyses to explore possible underlying sources of heterogeneity and effect modification. Subgroups investigated included drug class (omaveloxolone versus interferon-γ-1b versus other agents), treatment duration (short-term defined as less than 24 weeks versus long-term defined as 24 weeks or longer), and participant age group (pediatric population younger than 18 years versus adult population 18 years or older). Statistical tests for subgroup differences were conducted using chi-square tests for heterogeneity between subgroups. Multiple sensitivity analyses were performed to assess the robustness of primary findings, including restriction to studies at low risk of bias, exclusion of studies with imputed standard deviations, comparison of random-effects versus fixed-effect models, and leave-one-out meta-analyses by removing each study to evaluate the effect of individual studies on pooled estimates.

### 3.9. Assessment of Publication Bias

Formal assessment of publication bias (funnel-plot asymmetry, Egger’s and Begg’s tests, and trim-and-fill adjustment) requires approximately ten or more studies per outcome to be reliable. As no outcome met this threshold, publication bias was not formally assessed. We also assessed selective outcome reporting bias by comparing reported outcomes against registered protocols when available and evaluating whether studies with statistically significant results were more likely to be published.

### 3.10. Meta-Regression Modeling

We performed exploratory univariate meta-regression to examine whether treatment duration (weeks) was associated with the treatment effect on mFARS. Because each model rested on only two to three studies, these analyses were underpowered and are reported as hypothesis-generating only.

### 3.11. Multiple Testing Correction

To account for multiple comparisons across different outcome measures, we applied Bonferroni correction, false discovery rate (FDR) adjustment using the Benjamini–Hochberg method, and Q-value calculation using the Storey method. These corrections provided conservative estimates of statistical significance and controlled family-wise error rates across the multiple primary and secondary efficacy outcomes investigated.

### 3.12. Certainty of Evidence Assessment

We evaluated the certainty of evidence for primary and key secondary outcomes using the GRADE (Grading of Recommendations, Assessment, Development, and Evaluations) framework. Evidence from RCTs began as high certainty and was evaluated for possible downgrading based on five criteria: risk of bias (serious limitations in study design or execution), inconsistency (unexplained heterogeneity or variability in results), indirectness (indirect population, intervention, comparator, or outcome), imprecision (wide 95% CIs or small sample sizes), and publication bias (evidence of selective publication or reporting).

Evidence could also be upgraded based on a large magnitude of effect, dose-response gradient, or presence of plausible residual confounding that would reduce a demonstrated effect. The final certainty rating was categorized as high, moderate, low, or very low, reflecting our confidence that the true effect lies close to the estimate of effect.

### 3.13. Software and Statistical Packages

All statistical analyses were performed using RStudio statistical software with R version 4.4.2 with the meta package for meta-analysis, metafor package for meta-regression, and additional packages for forest plot generation, funnel plot construction, and sensitivity analyses. Statistical significance was defined as two-sided *p*-values less than 0.05, except where multiple testing corrections were applied. No validated minimal clinically important difference has been established for the mFARS or FARS; therefore, we report between-group differences as statistically significant rather than clinically meaningful, and interpret their magnitude relative to the natural-history progression rate of approximately 2 points per year on the mFARS.

## 4. Results

### 4.1. Study Selection and Characteristics

**Figure 1:**
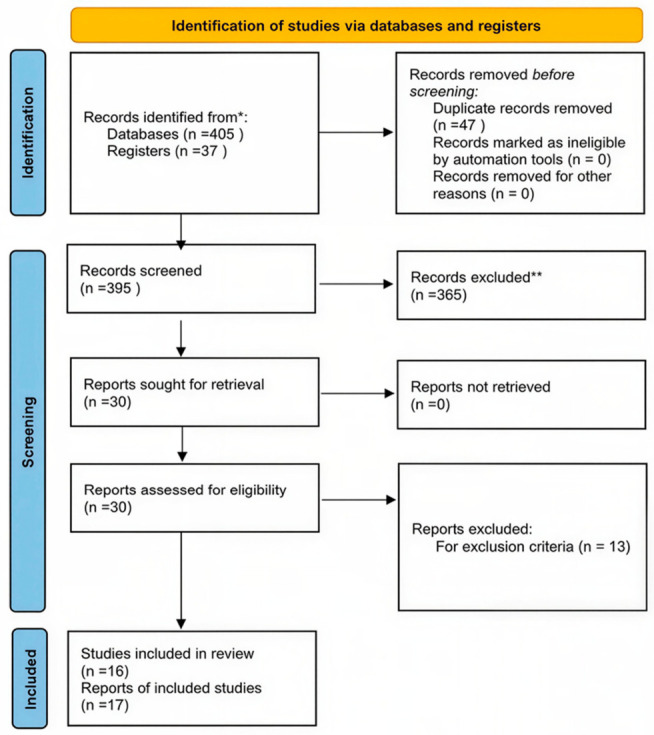
PRISMA 2020 flow diagram of study identification, screening, and inclusion. * Records were identified from three databases (PubMed, *n* = 103; Cochrane CENTRAL, *n* = 75; Google Scholar, *n* = 227) and two trial registers (ClinicalTrials.gov, *n* = 21; WHO ICTRP, *n* = 16). ** Records were excluded by two independent reviewers on the basis of title and abstract screening; no automation tools were used.

Our search retrieved all records up to 29 June 2025. A total of 442 records were identified from databases and trial registries. After removing 47 duplicates and screening titles/abstracts, 30 articles underwent full-text review. Sixteen studies (17 reports) met the criteria (Figure 1). Registry searches identified no additional eligible RCTs beyond those retrieved from PubMed. ClinicalTrials.gov yielded 21 records (zero new eligible RCTs), WHO ICTRP yielded 16 records (zero new), and Cochrane CENTRAL yielded 75 records (zero new). Google Scholar screening of the first 100 results for each of the four key drug searches (omaveloxolone, idebenone, vatiquinone, deferiprone) identified no additional eligible studies. Therefore, the review findings remained unchanged. The omaveloxolone trial (NCT02255435) contributed three reports—Part 1 (12-week dose-ranging), Part 2 (48-week primary efficacy), and a 72-week delayed-start extension. Part 1 and Part 2 were analyzed separately because they involved different populations and treatment durations.

Study characteristics are shown in Table 1 for the randomized controlled trials included in the quantitative synthesis and in Table 2 for the studies included in the narrative synthesis only. Designs included ten RCTs, two open-label RCT extensions, one delayed-start extension, one non-randomized comparative study, two single-arm dose-escalation studies, two single-arm pilots, and one phase I crossover trial. Phases included five phase I, five phase II, one phase III, and several with unspecified phase designation.

Sample sizes ranged from 9 to 103 participants, with 351 participants included in pooled safety analyses. Mean age ranged from 13.4 to 39.2 years; female representation ranged from 25% to 100%. Baseline disease severity showed presence of variability, with mean mFARS scores ranging from 40.5 to 44.4 points, FARS scores from 55.6 to 91.8 points, SARA scores from 12.0 to 36.0 points, and ICARS scores from 34.4 to 49.1 points where reported. Mean GAA repeat length in the shorter allele ranged from 568 to 1085 repeats among studies reporting this genetic characteristic.

Treatment duration ranged from single-dose pharmacokinetic studies to 72-week trials. The most frequently studied interventions were omaveloxolone (three reports from the MOXIe programme; Parts 1 and 2 enrolled non-overlapping cohorts and were counted as two studies, with the delayed-start extension counted as a further report of Part 2), idebenone (four studies), and interferon-γ-1b (two studies). Additional agents included leriglitazone, deferiprone, resveratrol, vatiquinone, RT001, darbepoetin alfa combinations, and histone deacetylase inhibitors.

### 4.2. Risk of Bias Assessment

Among the ten RCTs assessed with RoB 2.0, six (60%) were judged to be at low overall risk of bias, and four (40%) raised some concerns. Common issues were missing outcome data from attrition, particularly in longer-duration studies and concerns related to protocol deviations in the intervention delivery. Studies consistently judged to be at low risk included Lynch et al. 2021 [2], Lynch et al. 2019 [8], Lynch et al. 2019 [5], Lynch et al. 2010 [9], Zesiewicz et al. 2018 [12], and Soragni et al. 2014 [15]. These trials used centralized randomization, maintained high follow-up completeness, applied blinded assessment with validated scales, and reported all pre-registered outcomes. In contrast, Pandolfo et al. 2022 [7] raised concerns due to unequal dropout (23% vs. 8%) and one protocol deviation. Zesiewicz et al. 2018 [11] also had concerns despite adequate randomization and blinding; attrition reached 34% at 24 months, leaving substantial missing data not fully resolved by intention-to-treat analysis.

Non-randomized studies evaluated with ROBINS-I showed higher bias. Yiu et al. 2015 [14] was high risk due to non-random dose assignment, open-label design, lack of controls, and emphasis on positive secondary outcomes despite negative primary findings. Lynch et al. 2023 [6] and Meier et al. 2012 [17] (open-label RCT extensions) showed some concerns from loss of blinding and attrition, though preservation of randomization and use of objective scales partially mitigated risk. Single-arm studies [13,16,18,19,20] were not suitable for comparative bias tools but generally carried high risk due to open-label design, small cohorts, dropout rates up to 45%, unblinded outcome assessment, and reliance on historical or within-subject comparisons. Phase I safety/pharmacokinetic studies [10,15,19] were not assessed with clinical bias frameworks because of their purpose and methodology.

### 4.3. Primary Efficacy Outcomes

**Figure 2:**
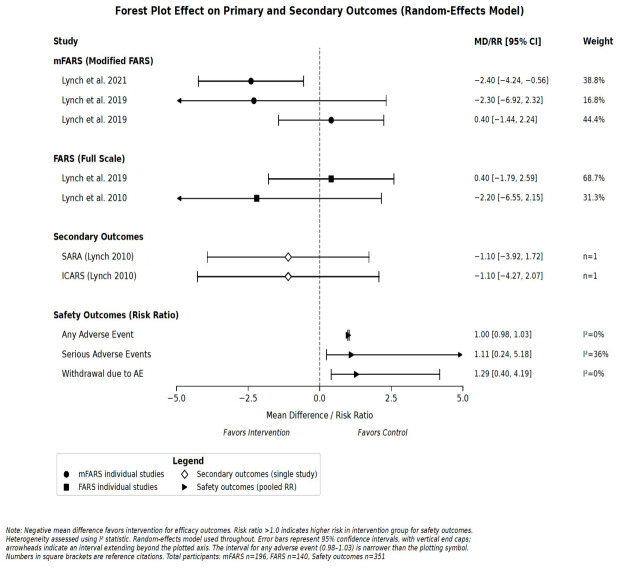
Forest Plot Effect on Primary and Secondary Outcomes (Random-Effects Model) (Lynch et al. 2021 [2]; Lynch et al. 2019 [5]; Lynch et al. 2019 [8]; Lynch et al. 2010 [9]).

The primary efficacy outcome mFARS is shown in the forest plot (Figure 2). Three RCTs reported mFARS, but because they evaluated pharmacologically distinct agents, we synthesized them by drug rather than as a single combined estimate.

For omaveloxolone, two reports involving different participant cohorts Lynch et al. 2021 [2], 150 mg daily over 48 weeks; Lynch et al. 2019 [5], 160 mg daily over 12 weeks) yielded a pooled mean difference of −2.39 points (95% CI −4.10 to −0.67; *p* < 0.05) with no heterogeneity (I^2^ = 0.0%, τ^2^ = 0.000), indicating a consistent improvement versus placebo. In the individual reports, Lynch et al. 2021 [2] (*n* = 82) showed a statistically significant improvement (MD −2.40; 95% CI −4.24 to −0.56; *p* = 0.014) and Lynch et al. 2019 [5] (*n* = 22) a numerically similar but non-significant effect (MD −2.30; 95% CI −6.92 to 2.32; *p* = 0.06), the wider confidence interval reflecting the smaller sample.

For interferon-γ-1b, the single eligible RCT Lynch et al. 2019 [8], 92 participants over 26 weeks) showed a small effect favoring control (MD +0.40; 95% CI −1.44 to 2.24; non-significant), indicating no benefit on mFARS.

For the full FARS scale, two RCTs reported data, but as they evaluated different agents we report them separately rather than pooled. Lynch et al. 2019 [8] (interferon-γ-1b) reported a mean difference of +0.40 points (95% CI −1.79 to 2.59), consistent with its mFARS result and indicating no benefit. Lynch et al. 2010 [9] high-dose idebenone, 2250 mg daily over 24 weeks) showed a numerically favorable but non-significant effect of −2.20 points (95% CI −6.55 to 2.15); standard deviations for this study were imputed using the Cochrane method (assumed correlation 0.5) owing to incomplete reporting in the original publication. Neither single-study estimate reached statistical significance.

### 4.4. Secondary Efficacy Outcomes

Only one RCT—Lynch et al. 2010 [9] (*n* = 48)—reported SARA, precluding meta-analysis. This study of high-dose idebenone demonstrated an MD of −1.10 points (95% CI −3.92 to 1.72; *p*-value was not significant) favoring intervention; however, with significant imprecision reflected in wide 95% CIs. Standard deviations for change scores were imputed using the Cochrane method with an assumed correlation coefficient of 0.5, introducing additional uncertainty into these estimates.

Similarly, for the ICARS scale, only one RCT [9] provided comparative data, showing an MD of −1.10 points (95% CI −4.27 to 2.07; *p*-value was not significant) favoring idebenone. Standard deviations were borrowed from Meier et al. 2012 [17] due to incomplete reporting in the original publication. One additional single-arm study [14] evaluated high-dose resveratrol 5 g daily over 12 weeks in 12 participants and reported a within-group improvement of −1.9 points (95% CI −3.1 to −0.8; *p* = 0.004) from baseline. However, this single-arm design without concurrent controls limits the interpretability of these findings, as the observed changes may reflect regression to the mean, practice effects, or natural disease variability rather than true treatment effects. Functional performance outcomes were inconsistent, with methodological heterogeneity that precluded proper pooling. The Nine-Hole Peg Test was reported in two trials, but different units of measurement prevented pooling: Lynch et al. 2021 [2] used reciprocal time (MD –0.0013; *p* = 0.18, non-dominant hand), while Lynch et al. 2019 [8] reported baseline values only, without change scores, making direct comparison impossible. The Timed 25-Foot Walk was similarly inconsistent—reciprocal time in Lynch et al. 2021 [2] (MD 0.0058; *p* = 0.46, favoring control) versus baseline-only reporting in Lynch et al. 2019 [8]. The use of reciprocal transformations to normalize distributions and address floor effects in these performance measures, while statistically appropriate, complicated interpretation and synthesis across studies.

### 4.5. Safety Outcomes

Five RCTs (*n* = 351; 200 intervention, 151 control) contributed pooled safety data. Adverse events were universal (100% in both groups); the pooled RR for any event was 1.00 (95% CI 0.98–1.03) with no heterogeneity (I^2^ = 0.0%, τ^2^ = 0.000, *p*-heterogeneity > 0.30). Common adverse events in intervention arms included headache (17–57%), nausea (33%) and elevated alanine aminotransferase (ALT) (37%) in Lynch et al. 2021 [2], fatigue (47%) and pyrexia (43%) in Lynch et al. 2019 [8], upper respiratory infections (40%) in Lynch et al. 2019 [5], and peripheral edema (73%) and weight gain (46%) in Pandolfo et al. 2022 [7].

Serious adverse events were uncommon (8/153 [5.2%] intervention vs. 5/113 [4.4%] control across four studies); the pooled RR was 1.11 (95% CI 0.24–5.18) with moderate heterogeneity (I^2^ = 36.0%, τ^2^ = 0.903), reflecting low event counts and imprecision. Reported serious events were heterogeneous (hospitalizations for progression, infections, cardiovascular events) with no clear drug-related pattern; no deaths occurred during treatment periods. Withdrawals due to adverse events were infrequent (9/176 [5.1%] vs. 4/127 [3.1%]; pooled RR 1.29, 95% CI 0.40–4.19) with minimal heterogeneity (I^2^ = 0.0%), and were mainly driven by elevated liver enzymes, GI intolerance, peripheral edema, and weight gain (notably in omaveloxolone and leriglitazone studies).

### 4.6. Certainty of Evidence

For the primary efficacy outcome (mFARS), GRADE was applied to the omaveloxolone estimate, the only drug-specific synthesis showing an effect. Beginning as high certainty (RCT evidence), it was downgraded one level for risk of bias (the estimate includes a small dose-ranging trial with some methodological concerns) and one level for imprecision (small total sample, below the approximate optimal information size of ~400, and reliance on a single trial program). No downgrade for inconsistency was applied, as the two omaveloxolone reports were homogeneous (I^2^ = 0.0%). The resulting certainty was low. For interferon-γ-1b and the remaining agents, no statistically significant effect was demonstrated and the supporting evidence was correspondingly limited.

For FARS, the certainty of evidence was very low. The two RCTs reporting FARS evaluated different agents (interferon-γ-1b [8] and idebenone [9]) and were not pooled; neither reached statistical significance. The rating was downgraded for risk of bias (imputed standard deviations in Lynch et al. 2010 [9]), serious imprecision (small single-study samples with wide confidence intervals), and a limited evidence base of one small trial per agent.

SARA change from baseline was rated as very low certainty based on one RCT (*n* = 48; Lynch et al. 2010 [9]), downgraded for risk of bias (imputed SDs) and for serious imprecision (wide CI −3.92 to 1.72). ICARS was also rated as very low certainty, coming from one small RCT plus a single-arm study and affected by high risk of bias, serious imprecision, and limited generalizability.

Safety findings were generally stronger. Any adverse event was rated as high certainty from five RCTs (*n* = 351) with no downgrades for risk of bias, inconsistency (I^2^ = 0.0%), indirectness, or imprecision (narrow CI 0.98–1.03), and publication bias was considered unlikely. In contrast, serious adverse events were rated as very low certainty (four trials, *n* = 266) due to moderate inconsistency (I^2^ = 36.0%) and serious imprecision (wide CI 0.24–5.18 and only 13 events). Withdrawals due to adverse events were rated as low certainty (four studies, *n* = 303): no downgrade for bias or inconsistency (I^2^ = 0.0%), but downgraded for imprecision (wide CI 0.40–4.19 and few events), limiting conclusions on tolerability.

### 4.7. Exploratory and Sensitivity Analyses

In subgroup analysis, the two omaveloxolone trials pooled to an MD of −2.39 points (95% CI −4.10 to −0.67; I^2^ = 0.0%) versus +0.40 for the single interferon-γ-1b trial, with a significant test for subgroup differences (*p*-interaction < 0.05). This reflects drug specificity rather than a class effect, and the parallel age difference cannot be interpreted independently because age was completely confounded with drug (the pediatric subgroup was the interferon-γ-1b study; the adult subgroup was omaveloxolone). Sensitivity analyses were consistent—restricted to low-risk studies, excluding imputed SDs, and comparing fixed- versus random-effects models did not change the conclusions, and leave-one-out analysis showed the signal was driven primarily by Lynch et al. 2021 [2]. Full results are shown in Table S6.

After correction for multiple comparisons (Bonferroni, Benjamini–Hochberg FDR, and Storey q-values), only omaveloxolone for mFARS and the single-arm resveratrol result for ICARS remained significant; the latter derives from a high-risk, uncontrolled study, confirming the efficacy signal is confined to omaveloxolone. Exploratory meta-regression restricted to omaveloxolone showed no association between treatment duration and mFARS (slope −0.003 points/week; *p* = 0.88) and was underpowered (two to three studies per model). These hypothesis-generating analyses are presented in full in the Supplement (Figures S1 and S2).

## 5. Discussion

This systematic review and meta-analysis provide a comprehensive evaluation of 16 pharmacological treatments for Friedreich’s ataxia (FRDA). Across the agents reviewed, omaveloxolone showed the most consistent signal of benefit and was the only therapy to reach statistical significance on the mFARS; given the low certainty of evidence and its reliance on a single trial program, it is best regarded as the most promising candidate to date rather than an established disease-modifying therapy [2,6]. The ~2.4-point difference is comparable to roughly one year of natural mFARS progression, although no formal minimal clinically important difference has been defined. Conversely, agents such as interferon-γ-1b showed limited therapeutic [8,13].

These findings align with the evolving therapeutic landscape for FRDA, highlighting a shift away from traditional, broad-spectrum agents. Older treatments, including conventional antioxidants like idebenone and deferiprone, yielded modest or inconsistent results on validated neurological scales [9,16,17,18,19] Similarly, mechanism-based agents like vatiquinone and RT001 primarily demonstrated changes in oxidative biomarkers without translating consistently into functional neurological gains [11,12,15,21].

Safety outcomes should be interpreted cautiously. Although overall adverse event rates were similar between intervention and control groups (RR 1.00; 95% CI 0.98–1.03), this finding should not be interpreted as evidence of a favorable safety profile. Serious adverse events were uncommon (8/153 vs. 5/113; RR 1.11, 95% CI 0.24–5.18), but the estimate was imprecise because of the low number of events. Withdrawals due to adverse events were also infrequent (9/176 vs. 4/127; RR 1.29, 95% CI 0.40–4.19) and were mainly associated with elevated liver enzymes, gastrointestinal intolerance, peripheral edema, and weight gain. In addition, follow-up durations were relatively short across most studies, limiting assessment of long-term safety. Therefore, while current evidence does not suggest a major increase in adverse events, confidence in the safety estimates remains limited.

The methodological rigor of this review was reinforced by adherence to PRISMA guidelines and PROSPERO registration [22]. However, limitations include the small sample sizes, variability in outcome measures, and short follow-up durations across the included studies, which may have reduced the sensitivity to detect genuine treatment effects. The absence of Embase searching, due to institutional access limitations, may represent a limitation; however, the comprehensive search of PubMed, CENTRAL, and major trial registries likely captured the key published RCTs in this rare disease. The predominance of industry-sponsored trials also raises the possibility of publication or reporting bias.

Future studies should standardize outcome tools, such as using mFARS, and extend follow-up periods beyond 48–72 weeks to fully capture long-term efficacy and safety. Implementing stratification based on genetic or biomarker profiles and exploring combined therapeutic strategies are essential steps to improve precision and accelerate the development of meaningful treatments for individuals with FRDA.

## 6. Conclusions

Friedreich’s ataxia (FRDA) is a challenging neurodegenerative disorder with limited pharmacologic treatment options. Among the therapies reviewed, omaveloxolone was the only agent to demonstrate a statistically significant improvement in neurological outcomes; however, this finding is based on very low-certainty evidence. Consequently, omaveloxolone may be the most promising current pharmacologic option, but its true efficacy remains uncertain and should be confirmed in larger, longer, and well-designed randomized controlled trials. Other pharmacologic agents showed no consistent evidence of benefit. Safety outcomes were broadly comparable between intervention and control groups; however, available safety evidence remains limited by imprecision, small sample sizes, and relatively short follow-up periods. Although no substantial increase in adverse events was observed, confidence in the safety estimates remains limited.

## Figures and Tables

**Table 1:** Characteristics of Randomized Controlled Trials Included in Quantitative Synthesis.

Study	Design	Phase	Intervention (Dose)	Comparator	Duration (Weeks)	Sample Size (N Randomized/N Analyzed)	Analysis Population	Age, Years Mean ± SD (Int/Ctrl)	Female, % (Int/Ctrl)	Disease Duration, Years Mean ± SD (Int/Ctrl)	Registration Number
Pandolfo et al. 2022 [7]	RCT	Phase II	Leriglitazone individualized daily oral dose	Placebo	48	39/34 (22 Int/12 Ctrl)	mITT	23.1 ± 9.8/25.8 ± 12.7	42.3/46.2	9.6 ± 5.1/12.3 ± 8.1	NCT03917225
Lynch et al. 2021 [2]	RCT	Phase II	Omaveloxolone 150 mg/day	Placebo	48	103/82 (40 Int/42 Ctrl)	FAS	24.2 ± 6.5/23.6 ± 7.8	60/33	4.8 ± 4.0/4.7 ± 4.7	NCT02255435
Lynch et al. 2019 [8]	RCT	NR	Interferon-γ-1b escalating to 100 µg/m^2^ 3×/wk	Placebo	26	92/92 (47 Int/45 Ctrl)	ITT	16.5 ± 4.4/16.1 ± 3.8	55.3/57.8	NR	NCT02593773
Lynch et al. 2019 [5]	RCT	Phase II (Part 1)	Omaveloxolone 160 mg/day †	Placebo	12	69/69 (12 at 160 mg/17 Ctrl)	ITT	25.9 ± 6.4/24.4 ± 6.7	52/59	11.1 ± 5.3/7.7 ± 3.5	NCT02255435
Lynch et al. 2010 [9]	RCT	Phase III	Idebenone 1350 or 2250 mg/day (high dose) †	Placebo	24	70/70 (24 high/24 placebo)	ITT	13.4 ± 3.0/13.7 ± 2.8	58.3/66.7	5.3 ± 2.8/5.9 ± 3.9	NCT00537680

Notes: † Multi-arm trial; data shown for highest dose arm compared to placebo; Abbreviations: RCT, randomized controlled trial; mITT, modified intent-to-treat; FAS, full analysis set; ITT, intent-to-treat; Int, intervention; Ctrl, control; NR, not reported;wk, week.

**Table 2:** Characteristics of Studies Included in Narrative Synthesis Only.

Study	Design	Phase	Intervention (Dose)	Comparator	Duration (Weeks)	Sample Size (N Randomized/N Analyzed)	Analysis Population	Age, Years Mean ± SD (Int/Ctrl)	Female, % (Int/Ctrl)	Disease Duration, Years Mean ± SD (Int/Ctrl)	Registration Number
Lee et al. 2024 [10]	RCT Crossover	Phase I	Vatiquinone 400 mg and 1400 mg single dose	Placebo	0.14 (1 day)	28/28	Crossover	NR/NR	NR/NR	NA (healthy volunteers)	NR
Lynch et al. 2023 [6]	Delayed-start extension	Phase II Ext	Omaveloxolone 150 mg/day	Delayed start (prev placebo)	72	82/73 (29 early/29 delayed at wk 72)	Modified	24.2 ± 6.5/23.6 ± 7.8	60/33	4.8 ± 4.0/4.7 ± 4.7	NCT02255435
Zesiewicz et al. 2018 [11]	RCT	NR	Vatiquinone 200 or 400 mg TID †	Placebo	24	63/61 (42 pooled Int/19 Ctrl)	ITT	29.1 (low), 28.7 (high)/29.7 ± 8.3	50/52.4	NR	NCT01728064
Zesiewicz et al. 2018 [12]	RCT	Phase I/II	RT001 1.8 or 9.0 g/day †	Non-deuterated ethyl linoleate	4	19/18 (12 pooled Int/6 Ctrl)	mITT	34 (18–48) ‡/37 (23–47) ‡	61.5/33.3	NR	NCT02445794
Marcotulli et al. 2016 [13]	Dose-escalation	Phase IIa	Interferon-γ-1b escalating (100, 150, 200 µg)	None (single arm)	5	9/9	NA	29.8 ± 6.0/NA	66.7/NA	NR	EudraCT2012–001881-14
Yiu et al. 2015 [14]	Non-randomized	NR	Resveratrol 5 g/day	Historical	12	27/24 (12 high-dose/NA)	NA	39.2 ± 7.7/NA	25/NA	19.4 ± 6.7/NA	NCT01339884
Soragni et al. 2014 [15]	Crossover	Phase I	RG2833 (HDAC inhibitor) 30–240 mg	Placebo	0.14 (1 day)	20/20	Crossover	30.0 ± 8.1/30.0 ± 8.1	59.1/59.1	NR	EudraCT 2011–000248-12
Arpa et al. 2013 [16]	Pilot study	NR	Darbepoetin alfa + Idebenone + Riboflavin	None (single arm)	32 (avg)	9/9	NA	28 ± 8/NA	100/NA	16.3 §/NA	NR
Meier et al. 2012 [17]	OLE of RCT	NR	Idebenone 1350 or 2250 mg/day	Historical	52	68/68 (22 high/46 low/placebo)	ITT	14.0 ± 2.72/NA	54.5/NA	NR	NCT00697073
Abbruzzese et al. 2011 [18]	Single-arm pilot	Phase II	Deferiprone 30 mg/kg/day	None (single arm)	52	6/6	NA	36.5 ± 17.1/NA	33.3/NA	NR	NTC00907283
DiProspero et al. 2007 [19]	Dose-escalation	Phase I	Idebenone up to 75 mg/kg (1a); 60 mg/kg/day (1b)	None (single arm)	4 (Phase 1b)	93/14 (Phase 1b)	NA	23.2 ± 12.4/NA	46.7/NA	NR	NCT00015808
Boddaert et al. 2007 [20]	Open trial	Phase I/II	Deferiprone 20–30 mg/kg/day	Historical	24	9/9	NA	17.6 ± 3.5/NA	77.8/NA	7.8 ± 2.4/NA	NCT00224640

Notes: † Multi-arm trial; data shown for highest dose arm compared to placebo; ‡ Median (range) reported; § Disease duration from diagnosis. Abbreviations: RCT, randomized controlled trial; Ext, extension; mITT, modified intent-to-treat; ITT, intent-to-treat; Int, intervention; Ctrl, control; NR, not reported; NA, not applicable; TID, three times daily; OLE, open-label extension; HDAC, histone deacetylase; SD, standard deviation; wk, week; avg, average; prev, previously.

## Data Availability

No new data were created or analyzed in this study. Data sharing is not applicable to this article.

## Supplementary Materials

The following supporting information can be downloaded at https://www.mdpi.com/article/10.3390/jcm15145707/s1. Table S1: Prisma 2020 checklist; Table S2: Primary Efficacy Outcomes for mFARS and FARS; Table S3: Risk of Bias Assessment for Included Studies; Table S4: Secondary Efficacy Outcomes (SARA, ICARS, and Performance Tests) with Individual Study Results; Table S5: Safety Outcomes and Adverse Events Results; Table S6: Subgroup and Sensitivity Analyses for Primary Outcome (mFARS); Table S7: Characteristics and Baseline Demographics of Included Studies; Table S8: GRADE Evidence Quality Assessment for Primary and Secondary Outcomes; Figure S1: Multiple Testing Correction Plot; Figure S2: Meta-Regression Modeling For Treatment Duration vs. Effect Size; Figure S3: Treatment Duration vs. Effect Size Across Ataxia Rating Scales.

## Abbreviations

The following abbreviations are used in this manuscript:

FRDA	Friedreich’s ataxia
AEs	Adverse events
RCTs	Randomized controlled trials
mFARS	Modified Friedreich Ataxia Rating Scale
FARS	Friedreich Ataxia Rating Scale
SARA	Scale for Assessment and Rating of Ataxia
MD	Mean difference
ALT	Alanine aminotransferase
MeSH	Medical Subject Headings
ATP	Adenosine triphosphate
CENTRAL	Cochrane Central Register of Controlled Trials
mITT	Modified intent-to-treat
CI	Confidence interval
Nrf2	Nuclear factor erythroid 2-related factor 2
CoQ10	Coenzyme Q10
OLE	Open-label extension
FDR	False discovery rate
PK	Pharmacokinetics
PRISMA	Preferred Reporting Items for Systematic Reviews and Meta-Analyses
PROSPERO	International Prospective Register of Systematic Reviews
FXN	Frataxin gene
RoB	Risk of Bias
GAA	Guanine–adenine–adenine repeat
ROBINS-I	Risk Of Bias In Non-randomized Studies of Interventions
GRADE	Grading of Recommendations, Assessment, Development, and Evaluations
RR	Risk ratio
HDAC	Histone deacetylase
SAE(s)	Serious adverse event(s)
ICARS	International Cooperative Ataxia Rating Scale
ICTRP	International Clinical Trials Registry Platform
SD	Standard deviation
ITT	Intent-to-treat
TID	Three times daily
TQT	Thorough QT
UPDRS	Unified Parkinson’s Disease Rating Scale
WHO	World Health Organization

## References

[1] Cook A., Giunti P. (2017). Friedreich’s ataxia: Clinical features, pathogenesis and management. Br. Med. Bull..

[2] Lynch D.R., Chin M.P., Delatycki M.B., Subramony S.H., Corti M., Hoyle J.C., Boesch S., Nachbauer W., Mariotti C., Mathews K.D. (2021). Safety and Efficacy of Omaveloxolone in Friedreich Ataxia (MOXIe Study). Ann. Neurol..

[3] Jain P., Badgujar L., Spoorendonk J., Buesch K. (2022). Clinical evidence of interventions assessed in Friedreich ataxia: A systematic review. Ther. Adv. Rare Dis..

[4] Scott V., Delatycki M.B., Tai G., Corben L.A. (2024). New and Emerging Drug and Gene Therapies for Friedreich Ataxia. CNS Drugs.

[5] Lynch D.R., Farmer J., Hauser L., Blair I.A., Wang Q.Q., Mesaros C., Snyder N., Boesch S., Chin M., Delatycki M.B. (2019). Safety, pharmacodynamics, and potential benefit of omaveloxolone in Friedreich ataxia. Ann. Clin. Transl. Neurol..

[6] Lynch D.R., Chin M.P., Boesch S., Delatycki M.B., Giunti P., Goldsberry A., Hoyle J.C., Mariotti C., Mathews K.D., Nachbauer W. (2023). Efficacy of Omaveloxolone in Friedreich’s Ataxia: Delayed-Start Analysis of the MOXIe Extension. Mov. Disord. Off. J. Mov. Disord. Soc..

[7] Pandolfo M., Reetz K., Darling A., Rodriguez de Rivera F.J., Henry P.G., Joers J., Lenglet C., Adanyeguh I., Deelchand D., Mochel F. (2022). Efficacy and Safety of Leriglitazone in Patients With Friedreich Ataxia: A Phase 2 Double-Blind, Randomized Controlled Trial (FRAMES). Neurol. Genet..

[8] Lynch D.R., Hauser L., McCormick A., Wells M., Dong Y.N., McCormack S., Schadt K., Perlman S., Subramony S.H., Mathews K.D. (2019). Randomized, double-blind, placebo-controlled study of interferon-γ 1b in Friedreich Ataxia. Ann. Clin. Transl. Neurol..

[9] Lynch D.R., Perlman S.L., Meier T. (2010). A phase 3, double-blind, placebo-controlled trial of idebenone in friedreich ataxia. Arch. Neurol..

[10] Lee L., Flach S., Xue H., Arivelu L., Golden L., Kong R., Darpo B. (2024). Lack of Concentration-QTc Relationship and Cardiac Risk With Vatiquinone Therapeutic and Supratherapeutic Doses. Clin. Pharmacol. Drug Dev..

[11] Zesiewicz T., Salemi J.L., Perlman S., Sullivan K.L., Shaw J.D., Huang Y., Isaacs C., Gooch C., Lynch D.R., Klein M.B. (2018). Double-blind, randomized and controlled trial of EPI-743 in Friedreich’s ataxia. Neurodegener. Dis. Manag..

[12] Zesiewicz T., Heerinckx F., De Jager R., Omidvar O., Kilpatrick M., Shaw J., Shchepinov M.S. (2018). Randomized, clinical trial of RT001: Early signals of efficacy in Friedreich’s ataxia. Mov. Disord..

[13] Marcotulli C., Fortuni S., Arcuri G., Tomassini B., Leonardi L., Pierelli F., Testi R., Casali C. (2016). GIFT-1, a phase IIa clinical trial to test the safety and efficacy of IFNγ administration in FRDA patients. Neurol. Sci. Off. J. Ital. Neurol. Soc. Ital. Soc. Clin. Neurophysiol..

[14] Yiu E.M., Tai G., Peverill R.E., Lee K.J., Croft K.D., Mori T.A., Scheiber-Mojdehkar B., Sturm B., Praschberger M., Vogel A.P. (2015). An open-label trial in Friedreich ataxia suggests clinical benefit with high-dose resveratrol, without effect on frataxin levels. J. Neurol..

[15] Soragni E., Miao W., Iudicello M., Jacoby D., De Mercanti S., Clerico M., Longo F., Piga A., Ku S., Campau E. (2014). Epigenetic therapy for Friedreich ataxia. Ann. Neurol..

[16] Arpa J., Sanz-Gallego I., Rodríguez-de-Rivera F.J., Domínguez-Melcón F.J., Prefasi D., Oliva-Navarro J., Moreno-Yangüela M., Pascual-Pascual S.I. (2013). Triple therapy with darbepoetin alfa, idebenone, and riboflavin in Friedreich’s ataxia: An open-label trial. Cerebellum.

[17] Meier T., Perlman S.L., Rummey C., Coppard N.J., Lynch D.R. (2012). Assessment of neurological efficacy of idebenone in pediatric patients with Friedreich’s ataxia: Data from a 6-month controlled study followed by a 12-month open-label extension study. J. Neurol..

[18] Abbruzzese G., Cossu G., Balocco M., Marchese R., Murgia D., Melis M., Galanello R., Barella S., Matta G., Ruffinengo U. (2011). A pilot trial of deferiprone for neurodegeneration with brain iron accumulation. Haematologica.

[19] Di Prospero N.A., Sumner C.J., Penzak S.R., Ravina B., Fischbeck K.H., Taylor J.P. (2007). Safety, tolerability, and pharmacokinetics of high-dose idebenone in patients with Friedreich ataxia. Arch. Neurol..

[20] Boddaert N., Le Quan Sang K.H., Rötig A., Leroy-Willig A., Gallet S., Brunelle F., Sidi D., Thalabard J.C., Munnich A., Cabantchik Z.I. (2007). Selective iron chelation in Friedreich ataxia: Biologic and clinical implications. Blood.

[21] Petrillo S., D’Amico J., La Rosa P., Bertini E.S., Piemonte F. (2019). Targeting Nrf2 for the Treatment of Friedreich’s Ataxia: A Comparison among Drugs. Int. J. Mol. Sci..

[22] Page M.J., McKenzie J.E., Bossuyt P.M., Boutron I., Hoffmann T.C., Mulrow C.D., Shamseer L., Tetzlaff J.M., Akl E.A., Brennan S.E. (2021). The PRISMA 2020 statement: An updated guideline for reporting systematic reviews. BMJ Clin. Res. Ed..

