# Supplementary material for: Evolution of Friedreich’s Ataxia Management Across Established and Emerging Therapies—Systematic Review and Meta-Analysis
Source: J Clin Med. 2026 Jul 21;15(14):5707. doi: 10.3390/jcm15145707 (PMC13413417; doi:10.3390/jcm15145707)
Supplement: Supplementary file 1 [file jcm-15-05707-s001.zip › jcm-4336955-supplementary.pdf]

Journal of
Clinical Medicine

Table S1. Prisma 2020 checklist

Section and Topic	Item #	Checklist item	Location
		**TITLE**	
Title	1	Identify the report as a systematic review.	1
		**ABSTRACT**	
Abstract	2	See the PRISMA 2020 for Abstracts checklist.	1
		**INTRODUCTION**	
Rationale	3	Describe the rationale for the review in the context of existing knowledge.	2
Objectives	4	Provide an explicit statement of the objective(s) or question(s) the review addresses.	2
		**METHODS**	
Eligibility criteria	5	Specify the inclusion and exclusion criteria for the review and how studies were grouped for the syntheses.	2
Information sources	6	Specify all databases, registers, websites, organisations, reference lists and other sources searched or consulted to identify studies. Specify the date when each source was last searched or consulted.	2
Search strategy	7	Present the full search strategies for all databases, registers and websites, including any filters and limits used.	3
Selection process	8	Specify the methods used to decide whether a study met the inclusion criteria of the review, including how many reviewers screened each record and each report retrieved, whether they worked independently, and if applicable, details of automation tools used in the process.	3
Data collection process	9	Specify the methods used to collect data from reports, including how many reviewers collected data from each report, whether they worked independently, any processes for obtaining or confirming data from study investigators, and if applicable, details of automation tools used in the process.	3
Data items	10a	List and define all outcomes for which data were sought. Specify whether all results that were compatible with each outcome domain in each study were sought (e.g. for all measures, time points, analyses), and if not, the methods used to decide which results to collect.	4
	10b	List and define all other variables for which data were sought (e.g. participant and intervention characteristics, funding sources). Describe any assumptions made about any missing or unclear information.	4
Study risk of bias assessment	11	Specify the methods used to assess risk of bias in the included studies, including details of the tool(s) used, how many reviewers assessed each study and whether they worked independently, and if applicable, details of automation tools used in the process.	4
Effect measures	12	Specify for each outcome the effect measure(s) (e.g. risk ratio, mean difference) used in the synthesis or presentation of results.	4
Synthesis methods	13a	Describe the processes used to decide which studies were eligible for each synthesis (e.g. tabulating the study intervention characteristics and comparing against the planned groups for each synthesis (item #5)).	5
	13b	Describe any methods required to prepare the data for presentation or synthesis, such as handling of missing summary statistics, or data conversions.	5
	13c	Describe any methods used to tabulate or visually display results of individual studies and syntheses.	5
	13d	Describe any methods used to synthesize results and provide a rationale for the choice(s). If meta-analysis was performed, describe the model(s), method(s) to identify the presence and extent of statistical heterogeneity, and software package(s) used.	5
	13e	Describe any methods used to explore possible causes of heterogeneity among study results (e.g. subgroup analysis, meta-regression).	5
	13f	Describe any sensitivity analyses conducted to assess robustness of the synthesized results.	5

Section and Topic	Item #	Checklist item	Location
Reporting bias assessment	14	Describe any methods used to assess risk of bias due to missing results in a synthesis (arising from reporting biases).	5
Certainty assessment	15	Describe any methods used to assess certainty (or confidence) in the body of evidence for an outcome.	5
**RESULTS**			
Study selection	16a	Describe the results of the search and selection process, from the number of records identified in the search to the number of studies included in the review, ideally using a flow diagram.	6
	16b	Cite studies that might appear to meet the inclusion criteria, but which were excluded, and explain why they were excluded.	6
Study characteristics	17	Cite each included study and present its characteristics.	7
Risk of bias in studies	18	Present assessments of risk of bias for each included study.	10
Results of individual studies	19	For all outcomes, present, for each study: (a) summary statistics for each group (where appropriate) and (b) an effect estimate and its precision (e.g. confidence/credible interval), ideally using structured tables or plots.	11
Results of syntheses	20a	For each synthesis, briefly summarise the characteristics and risk of bias among contributing studies.	16
	20b	Present results of all statistical syntheses conducted. If meta-analysis was done, present for each the summary estimate and its precision (e.g. confidence/credible interval) and measures of statistical heterogeneity. If comparing groups, describe the direction of the effect.	14
	20c	Present results of all investigations of possible causes of heterogeneity among study results.	15
	20d	Present results of all sensitivity analyses conducted to assess the robustness of the synthesized results.	14
Reporting biases	21	Present assessments of risk of bias due to missing results (arising from reporting biases) for each synthesis assessed.	16
Certainty of evidence	22	Present assessments of certainty (or confidence) in the body of evidence for each outcome assessed.	13
**DISCUSSION**			
Discussion	23a	Provide a general interpretation of the results in the context of other evidence.	17
	23b	Discuss any limitations of the evidence included in the review.	17
	23c	Discuss any limitations of the review processes used.	17
	23d	Discuss implications of the results for practice, policy, and future research.	17
**OTHER INFORMATION**			
Registration and protocol	24a	Provide registration information for the review, including register name and registration number, or state that the review was not registered.	2
	24b	Indicate where the review protocol can be accessed, or state that a protocol was not prepared.	2
	24c	Describe and explain any amendments to information provided at registration or in the protocol.	2
Support	25	Describe sources of financial or non-financial support for the review, and the role of the funders or sponsors in the review.	35
Competing interests	26	Declare any competing interests of review authors.	35
Availability of data, code and other materials	27	Report which of the following are publicly available and where they can be found: template data collection forms; data extracted from included studies; data used for all analyses; analytic code; any other materials used in the review.	35

Table S2. Primary Efficacy Outcomes for mFARS and FARS.

Study	Outcome Scale	Intervention Group			Control Group			Between-Group Comparison				Direction	Data Quality
		Baseline Mean ± SD	Change Mean ± SD	Number	Baseline Mean ± SD	Change Mean ± SD	Number	MD	95% CI	P-value	Weight (%)		
Lynch et al. 2021	mFARS	40.9 ± 10.4	-1.55 ± 4.36	40	38.8 ± 11.0	0.85 ± 4.15	42	-2.40	(-4.24, -0.56)	0.014	38.8	Favors intervention	SD calculated from SE†
Lynch et al. 2019b	mFARS	40.5 ± 10.0	-3.8 ± 4.22	12	41.3 ± 12.0	-1.5 ± 6.39	10	-2.30	(-6.92, 2.32)	0.06	16.8	Favors intervention	160mg dose; SD calculated from SE†
Lynch et al. 2019a	mFARS	44.4 ± 11.9	-0.6 ± 4.6	47	44.1 ± 10.0	-1.0 ± 4.4	45	0.40	(-1.44, 2.24)	NS	44.4	Favors control	Interferon -γ 1b
**POOLED ESTIMATE**	**mFARS**	—	—	**99**	—	—	**97**	**-1.21**	**(-3.35, 0.94)**	**0.27**	—	**Favors intervention**	**Random-effects; I^2 = 57.7%; τ^2 = 1.970; P-het = 0.10**
Lynch et al. 2019a	FARS	55.6 ± 13.8	-0.2 ± 5.5	47	55.7 ± 10.8	-0.6 ± 5.2	45	0.40	(-1.79, 2.59)	NS	68.7	Favors control	Interferon -γ 1b
Lynch et al. 2010	FARS	56.5 ± 11.6	-1.6 ± 7.82	24	55.9 ± 10.4	0.6 ± 7.55	24	-2.20	(-6.55, 2.15)	NS	31.3	Favors intervention	High-dose idebenone; SD imputed‡
**POOLED ESTIMATE**	**FARS**	—	—	**71**	—	—	**69**	**-0.19**	**(-2.33, 1.95)**	**0.86**	—	**No difference**	**Random-effects; I^2 = 8.8%; τ^2 = 0.296; P-het >0.30**

Notes: *† SD calculated from standard error using formula: SD = SE × √n; ‡ SD imputed using Cochrane method with assumed correlation coefficient r = 0.5 (sensitivity analysis performed with r = 0.4-0.7).* ***Abbreviations:*** *mFARS, modified Friedreich Ataxia Rating Scale; FARS, Friedreich Ataxia Rating Scale; MD, mean difference (negative values favor intervention); CI, confidence interval; NS, not significant (P >0.05); I^2, inconsistency index (percentage of variability due to heterogeneity); τ^2, between-study variance; P-het, p-value for heterogeneity test; SD, standard deviation; SE, standard error; —, not applicable.*

Table S3. Risk of Bias Assessment for Included Studies.

Study	Study Design	Assessment Tool	Domain 1: Randomization Process	Domain 2: Deviations from Interventions	Domain 3: Missing Outcome Data	Domain 4: Outcome Measurement	Domain 5: Selective Reporting	Overall Risk of Bias	Funding Source
			Rating / Support	Rating / Support	Rating / Support	Rating / Support	Rating / Support		
Lee et al. 2024	Phase I TQT Crossover	RoB 2.0	Low / Crossover RCT; randomized sequence; adequate allocation	Low / Complete crossover; no dropouts (0%); healthy volunteers	Low / 28/28 completed (100%); complete data	Low / Double-blind; objective ECG measurements	Low / TQT study; regulatory requirement; all outcomes reported	N/A (Phase I in healthy volunteers)	Industry
Lynch et al. 2023	Delayed-start extension	ROBINS-I	Low / Extension of RCT; original randomization preserved	Some concerns / Open-label extension; 13.4% dropout	Some concerns / 73/82 enrolled; some attrition affects delayed-start analysis	Some concerns / Open-label but objective scales; delayed-start design reduces bias	Low / Extension of NCT02255435; pre-specified analysis	Some concerns	Industry
Pandolfo et al. 2022	RCT	RoB 2.0	Low / 2:1 randomization using IRT system; adequate concealment	Some concerns / 1 control patient excluded for protocol deviation; mITT used	Some concerns / 17.9% dropout; imbalanced (6/26 Int vs 1/13 Ctrl)	Low / Double-blind maintained; objective assessments	Low / Pre-registered NCT03917225; primary + clinical outcomes reported	Some concerns	Industry
Lynch et al. 2021	RCT	RoB 2.0	Low / Centralized IWRS; block randomization stratified by pes cavus	Low / FAS analysis; minimal discontinuations (8.7%); adherence monitored	Low / MMRM analysis; dropout balanced (4 Int vs 5 Ctrl); <10% overall	Low / Double-blind; assessors blinded to lab values; objective scale	Low / Pre-registered NCT02255435; primary outcome matched protocol; FDA approved	Low risk	Industry
Lynch et al. 2019a	RCT	RoB 2.0	Low / Randomized 1:1; stratification by age; allocation concealment adequate	Low / ITT analysis; no dropouts (1.1%); minimal protocol deviations	Low / Complete data for 92/92 (100%); no missing outcome data	Low / Double-blind; objective rating scales; trained assessors	Low / Pre-registered NCT02593773; all outcomes reported as specified	Low risk	Industry
Lynch et al. 2019b	RoB 2.0	Low / Dose-ranging RCT; central randomization; appropriate allocation	Low / ITT analysis; 2.9% dropout; no significant deviations	Low / Minimal missing data; 67/69 completed (97.1%)	Low / Double-blind; objective scales; blinded assessment	Low / Part 1 of NCT02255435; outcomes reported as planned	Low risk	Industry	-
Zesiewicz et al. 2018a	RCT	RoB 2.0	Low / Multi-center RCT; central randomization	Some concerns / 34% dropout by 24 months; ITT maintained but high attrition	High risk / High dropout (34%); missing data for many outcomes	Low / Double-blind; objective measurements	Low / Pre-registered NCT01728064; primary outcome reported	Some concerns	Industry

Study	Study Design	Assessment Tool	Domain 1: Randomization Process	Domain 2: Deviations from Interventions	Domain 3: Missing Outcome Data	Domain 4: Outcome Measurement	Domain 5: Selective Reporting	Overall Risk of Bias	Funding Source
			Rating / Support	Rating / Support	Rating / Support	Rating / Support	Rating / Support		
Zesiewicz et al. 2018b	RCT	RoB 2.0	Low / Randomized to 3 groups; adequate allocation	Low / mITT analysis; 5.3% dropout; minimal deviations	Low / 18/19 analyzed (94.7%); minimal missing data	Low / Double-blind; active comparator control	Some concerns / Pre-registered NCT02445794; 4-week exploratory study	Low risk	Industry
Marcotulli et al. 2016	Single-arm dose-escalation	N/A	N/A / Single-arm; no randomization or control	N/A / Phase IIa safety study; all received intervention	Low / 9/9 completed (100%); no missing data	Some concerns / Open-label; primary outcome safety; clinical scales secondary	Low / Safety study; all safety outcomes reported	N/A (Safety study)	Mixed
Yiu et al. 2015	Non-randomized	ROBINS-I	High risk / Non-randomized assignment to dose groups; selection bias likely	High risk / Open-label; no control group; within-subject comparison only	Some concerns / 11% dropout (3/27); completers analyzed	High risk / Open-label; assessors not blinded; subjective outcomes	Some concerns / Primary outcome (frataxin) negative; emphasized positive secondary outcomes	High risk	Public
Soragni et al. 2014	Phase I crossover	RoB 2.0	Low / Crossover design; randomized sequence; double-blind cohorts 3&4	Low / Complete crossover; ITT; no dropouts (0%)	Low / 20/20 completed (100%); complete data	Low / Double-blind (cohorts 3&4); biomarker outcomes	Low / Pre-registered EudraCT 2011-000248-12; PK/PD outcomes reported	N/A (Phase I PK/PD)	Industry
Arpa et al. 2013	Single-arm pilot	N/A	N/A / Single-arm pilot; no randomization or control	N/A / Pilot study; long follow-up (avg 32 weeks)	High risk / 22% dropout (2/9); high for small study	High risk / Open-label; no control; comparison to historical data only	Some concerns / Not pre-registered; small pilot; descriptive analysis	High risk	Public
Meier et al. 2012	Open-label extension	ROBINS-I	Some concerns / Extension of RCT but comparison to historical controls	Some concerns / Open-label; ITT maintained; 13.2% dropout	Some concerns / 68/68 analyzed but high attrition from original study	Some concerns / Open-label; objective scales but no concurrent control	Low / Extension of NCT00697073; analysis plan specified	Some concerns	Industry
Abbruzzese et al. 2011	Single-arm pilot	N/A	N/A / Single-arm pilot in NBIA patients (not pure FRDA)	N/A / Mixed population; 4/6 PKAN patients; safety focus	High risk / 45% dropout from original 11 enrolled; only 6 analyzed	High risk / Open-label; no control; mixed outcomes (UPDRS, ICARS)	Some concerns / Pre-registered NTC00907283; pilot study	High risk	Mixed
Lynch et al. 2010	RCT	RoB 2.0	Low / Central block randomizatio	Low / ITT analysis; no dropouts	Low / Complete data for all 70	Low / Double-blind;	Low / Pre-registered NCT0053768	Low risk	Industry

Study	Study Design	Assessment Tool	Domain 1: Randomization Process	Domain 2: Deviations from Interventions	Domain 3: Missing Outcome Data	Domain 4: Outcome Measurement	Domain 5: Selective Reporting	Overall Risk of Bias	Funding Source
			Rating / Support	Rating / Support	Rating / Support	Rating / Support	Rating / Support		
			n; adequate concealment documented	(0%); excellent adherence	participants; no attrition	validated scales; trained raters	0; primary outcome (ICARS) reported		
DiProspero et al. 2007	Phase I dose-escalation	N/A	N/A / Phase I safety/PK study; no efficacy endpoints	N/A / Safety and tolerability primary; dose escalation design	Some concerns / Phase 1b: 14/93 analyzed; many excluded for dose escalation	N/A / Safety study; no formal efficacy scales	Low / Pre-registered NCT00015808; safety outcomes reported	N/A (Phase I safety)	Mixed
Boddaert et al. 2007	Single-arm	N/A	N/A / Single-arm; comparison to historical controls	N/A / Open-label safety/efficacy study	High risk / 31% dropout (4/13 initially enrolled); high attrition	High risk / Open-label; primary outcome imaging; clinical scales secondary	Some concerns / Pre-registered NCT00224640; primary outcome reported	High risk	Mixed

Notes: *RoB 2.0, Cochrane Risk of Bias tool version 2.0 for randomized trials; ROBINS-I, Risk Of Bias In Non-randomized Studies of Interventions; N/A, not applicable (Phase I safety studies and single-arm studies cannot be assessed with comparative bias tools); IRT, interactive response technology; IWRS, interactive web response system; ITT, intention-to-treat; mITT, modified intention-to-treat; FAS, full analysis set; MMRM, mixed model for repeated measures; TQT, thorough QT study; PK, pharmacokinetics; PD, pharmacodynamics; NBIA, neurodegeneration with brain iron accumulation; PKAN, pantothenate kinase-associated neurodegeneration.*

Table S4. Secondary Efficacy Outcomes (SARA, ICARS, and Performance Tests) with Individual Study Results.

Study	Outcome Scale	Intervention Group			Control Group			Between-Group Comparison				Direction	Data Quality
		Baseline Mean ± SD	Change Mean ± SD	Number	Baseline Mean ± SD	Change Mean ± SD	Number	MD	95% CI	P-value	Weight (%)		
Lynch et al. 2010	SARA	36.0 ± 7.1	-2.4 ± 5.02	24	35.6 ± 7.0	-1.3 ± 4.95	24	-1.10	(-3.92, 1.72)	NS	—	Favors intervention	High-dose idebenone; SD imputed‡
**META-ANALYSIS**	**SARA**	—	—	—	—	—	—	**Not feasible**	—	—	—	—	**Insufficient RCTs (n=1)**
Lynch et al. 2010	ICARS	36.0 ± 7.1	-2.4 ± 5.60	24	35.6 ± 7.0	-1.3 ± 5.60	24	-1.10	(-4.27, 2.07)	NS	—	Favors intervention	High-dose idebenone; SD borrowed ‡
Yiu et al. 2015§	ICARS	49.1 ± 17.5	-1.9 ± 3.1	12	—	—	—	-1.9	(-3.1, -0.8)	0.004	—	Favors intervention	High-dose resveratrol; single-arm
**META-ANALYSIS**	**ICARS**	—	—	—	—	—	—	**Not feasible**	—	—	—	—	**Insufficient RCTs (n=1)**
Lynch et al. 2021	9-Hole Peg Test¶	—	-0.0014 ± 0.0044	40	—	-0.0001 ± 0.0039	42	-0.0013	—	0.18	—	Favors intervention	Non-dominant hand; reciprocal time
Lynch et al. 2019a	9-Hole Peg Test¶	0.0190 ± 0.0065	NR	47	0.0200 ± 0.0055	NR	45	NR	—	NR	—	—	Interferon-γ 1b; baseline only
**META-ANALYSIS**	**9-Hole Peg Test**	—	—	—	—	—	—	**Not feasible**	—	—	—	—	**Unit inconsistency (reciprocal time)**
Lynch et al. 2021	Timed 25FW¶	—	-0.0169 ± 0.0353	40	—	-0.0226 ± 0.0344	42	0.0058	—	0.46	—	Favors control	Reciprocal time
Lynch et al. 2019a	Timed 25FW¶	0.111 ± 0.074	NR	47	0.118 ± 0.065	NR	45	NR	—	NR	—	—	Interferon-γ 1b; baseline only
**META-ANALYSIS**	**Timed 25FW**	—	—	—	—	—	—	**Not feasible**	—	—	—	—	**Unit inconsistency (reciprocal time)**

Notes: *‡ SD imputed using Cochrane method with assumed correlation coefficient r = 0.5 (SARA) or borrowed from Meier et al. 2012 (ICARS); § Single-arm study comparing baseline to endpoint within same patients; ¶ Performance tests reported as reciprocal time (sec^{-1}) which cannot be directly pooled with studies reporting actual time (sec).* ***Abbreviations:*** *SARA, Scale for Assessment and Rating of Ataxia; ICARS, International Cooperative Ataxia Rating Scale; Timed 25FW, Timed 25-Foot Walk; MD, mean difference (negative values favor intervention for ataxia scales; positive values favor intervention for performance tests); CI, confidence interval; NS, not significant (P >0.05); NR, not reported; SD, standard deviation; RCT, randomized controlled trial; —, not applicable.*

Table S5. Safety Outcomes and Adverse Events Results.

Study	Total N (Int / Ctrl)	Any Adverse Event n/N (%)		Serious Adverse Events n/N (%)		Withdrawals Due to AEs n/N (%)		Deaths n		Between-Group Comparison				
		Intervention	Control	Intervention	Control	Intervention	Control	Int	Ctrl	Outcome	RR	95% CI	Weight (%)	Common AEs (Intervention)
Lynch et al. 2021	51 / 52	51/51 (100)	52/52 (100)	5/51 (9.8)	3/52 (5.8)	4/51 (7.8)	2/52 (3.8)	0	0	Any AE	1.00	(0.96, 1.04)	45.6	Headache (37%), Nausea (33%), ALT↑ (37%)
Lynch et al. 2019a	47 / 45	47/47 (100)	45/45 (100)	NR	NR	0/47 (0)	1/45 (2.2)	0	0	Any AE	1.00	(0.96, 1.04)	36.5	Headache (57%), Fatigue (47%), Pyrexia (43%)
Lynch et al. 2019b	52 / 17	52/52 (100)	17/17 (100)	0/52 (0)	2/17 (11.8)	1/52 (1.9)	1/17 (5.9)	0	0	Any AE	1.02	(0.94, 1.11)	9.5	Upper respiratory infection (40%), Headache (17%)
Lynch et al. 2010	24 / 24	24/24 (100)	24/24 (100)	2/24 (8.3)	0/24 (0)	NR	NR	0	0	Any AE	1.00	(0.92, 1.08)	10.2	GI irritations (58% Int vs 42% Ctrl)
Pandolfo et al. 2022	26 / 13	26/26 (100)	13/13 (100)	1/26 (3.8)	0/13 (0)	4/26 (15.4)	0/13 (0)	0	0	Any AE	1.02	(0.91, 1.14)	5.0	Peripheral edema (73%), Weight gain (46%)
**POOLED ESTIMATE**	**200 / 151**	**200/200 (100)**	**151/151 (100)**	**8/153 (5.2)**	**5/113 (4.4)**	**9/176 (5.1)**	**4/127 (3.1)**	**0**	**0**	**Any AE**	**1.00**	**(0.98, 1.03)**	—	**I^2 = 0.0%; τ^2 = 0.000; P-het >0.30**
										**SAE**	**1.11**	**(0.24, 5.18)**	—	**I^2 = 36.0%; τ^2 = 0.903; P-het >0.30**
										**Withdrawal**	**1.29**	**(0.40, 4.19)**	—	**I^2 = 0.0%; τ^2 = 0.000; P-het >0.30**

Abbreviations: *AE, adverse event; SAE, serious adverse event; Int, intervention; Ctrl, control; RR, risk ratio (values >1.0 indicate higher risk in intervention group); CI, confidence interval; NR, not reported; ALT, alanine aminotransferase; GI, gastrointestinal;* I^2*, inconsistency index;* τ^2*, between-study variance; P-het, p-value for heterogeneity test; —, not applicable.*

Table S6. Subgroup and Sensitivity Analyses for Primary Outcome (mFARS).

Analysis Type	Subgroup / Scenario	Number of Studies	Total N (Int / Ctrl)	Pooled MD	95% CI	P-value	I² (%)	τ²	Interpretation
**PRIMARY ANALYSIS**	All studies (random-effects)	3	99 / 97	-1.21	(-3.35, 0.94)	NS	57.7	1.970	Favors intervention, not significant
**SUBGROUP: Drug Class**	Omaveloxolone	2	52 / 52	-2.39	(-4.10, -0.67)	<0.05	0.0	0.000	Favors intervention, significant
	Interferon-γ 1b	1	47 / 45	0.40	(-1.44, 2.24)	NS	—	—	Single study; favors control
	**Test for subgroup differences**	—	—	**Δ = -2.79**	—	**P-int <0.05**	—	—	**Significant difference between drug classes**
**SUBGROUP: Duration**	Short-term (<24 weeks)	1	12 / 10	-2.30	(-6.92, 2.32)	NS	—	—	Single study; omaveloxolone 12 wk
	Long-term (≥24 weeks)	2	87 / 87	-1.00	(-3.74, 1.74)	NS	77.5	2.120	Substantial heterogeneity
	**Test for subgroup differences**	—	—	—	—	**P-int NS**	—	—	**No significant difference by duration**
**SUBGROUP: Age**	Pediatric (<18 years)	1	47 / 45	0.40	(-1.44, 2.24)	NS	—	—	Single study; interferon in children
	Adult (≥18 years)	2	52 / 52	-2.39	(-4.10, -0.67)	<0.05	0.0	0.000	Favors intervention, significant
	**Test for subgroup differences**	—	—	**Δ = -2.79**	—	**P-int <0.05**	—	—	Age subgroup confounded by treatment type
**SENSITIVITY: Study Quality**	Low risk of bias only	3	99 / 97	-1.21	(-3.35, 0.94)	NS	57.7	1.970	All studies low risk; same as primary
**SENSITIVITY: Imputed Data**	Excluding studies with imputed SD	3	99 / 97	-1.21	(-3.35, 0.94)	NS	57.7	1.970	No imputed data in mFARS studies
**SENSITIVITY: Model**	Fixed-effect model	3	99 / 97	-1.35	(-2.51, -0.18)	<0.05	57.7	—	Fixed-effect shows significant result
**LEAVE-ONE-OUT**	Excluding Lynch et al. 2021	2	59 / 55	-0.08	(-2.11, 1.94)	NS	54.2	1.200	Result becomes non-significant
	Excluding Lynch et al. 2019b	2	87 / 87	-1.00	(-3.74, 1.74)	NS	77.5	2.120	Heterogeneity increases
	Excluding Lynch et al. 2019a	2	52 / 52	-2.39	(-4.10, -0.67)	<0.05	0.0	0.000	Result becomes significant

Abbreviations: *MD, mean difference (negative values favor intervention); CI, confidence interval; NS, not significant (P >0.05); I², inconsistency index; τ², between-study variance; P-int, p-value for interaction test; Δ, difference between subgroups; —, not applicable (single study or fixed-effect model).*

Table S7. Characteristics and Baseline Demographics of Included Studies.

Study	Design	Phase	Intervention (Dose)	Comparator	Duration (weeks)	Sample Size (N randomized / N analyzed)	Analysis Population	Age, years Mean ± SD (Int / Ctrl)	Female, % (Int / Ctrl)	Disease Duration, years Mean ± SD (Int / Ctrl)	GAA Repeats (shorter allele) Mean ± SD	Baseline Disease Severity Mean ± SD	Registration Number
Lee et al. 2024	RCT Crossover	Phase I	Vatiquinone 400 mg and 1400 mg single dose	Placebo	0.14 (1 day)	28 / 28	Crossover	NR / NR	NR / NR	NA (healthy volunteers)	NA	NA (TQT study, healthy volunteers)	NR
Lynch et al. 2023	Delayed-start extension	Phase II Ext	Omaveloxolone 150 mg/day	Delayed start (prev placebo)	72	82 / 73 (29 early / 29 delayed at wk 72)	Modified	24.2 ± 6.5 / 23.6 ± 7.8	60 / 33	4.8 ± 4.0 / 4.7 ± 4.7	739.2 ± 214.9 / 693.8 ± 277.2	mFARS: 40.9 ± 10.4 / 38.8 ± 11.0	NCT02255435
Pandolfo et al. 2022	RCT	Phase II	Leriglitazone individualized daily oral dose	Placebo	48	39 / 34 (22 Int / 12 Ctrl)	mITT	23.1 ± 9.8 / 25.8 ± 12.7	42.3 / 46.2	9.6 ± 5.1 / 12.3 ± 8.1	NR	SARA: 12.9 ± 4.9 / 12.0 ± 4.3	NCT03917225
Lynch et al. 2021	RCT	Phase II	Omaveloxolone 150 mg/day	Placebo	48	103 / 82 (40 Int / 42 Ctrl)	FAS	24.2 ± 6.5 / 23.6 ± 7.8	60 / 33	4.8 ± 4.0 / 4.7 ± 4.7	739.2 ± 214.9 / 693.8 ± 277.2	mFARS: 40.9 ± 10.4 / 38.8 ± 11.0	NCT02255435
Lynch et al. 2019a	RCT	NR	Interferon-γ 1b escalating to 100 $\mu g/m^2$ 3×/wk	Placebo	26	92 / 92 (47 Int / 45 Ctrl)	ITT	16.5 ± 4.4 / 16.1 ± 3.8	55.3 / 57.8	NR	706 ± 166 / 711 ± 224	mFARS: 44.4 ± 11.9 / 44.1 ± 10.0; FARS: 55.6 ± 13.8 / 55.7 ± 10.8	NCT02593773
Lynch et al. 2019b	RCT	Phase II (Part 1)	Omaveloxolone 160 mg/day†	Placebo	12	69 / 69 (12 at 160mg / 17 Ctrl)	ITT	25.9 ± 6.4 / 24.4 ± 6.7	52 / 59	11.1 ± 5.3 / 7.7 ± 3.5	863 ± 278 / 700 ± 277	mFARS: 40.5 ± 10.0 / 41.3 ± 12.0	NCT02255435
Petrillo et al. 2019¶	In vitro	NA	Multiple NRF2 activators	None	0.001 (24 h)	0 / NA	NA	NA	NA	NA	NA	NA (in vitro study)	NA
Zesiewicz et al. 2018a	RCT	NR	EPI-743 (vatiquinone) 200 or 400 mg TID†	Placebo	24	63 / 61 (42 pooled Int / 19 Ctrl)	ITT	29.1 (low), 28.7 (high) / 29.7 ± 8.3	50 / 52.4	NR	NR	FARS-Neuro: 62.4 (low), 70.6 (high) / 55.9 ± 14.5	NCT01728064
Zesiewicz et al. 2018b	RCT	Phase I/II	RT001 1.8 or 9.0 g/day†	Non-deuterated ethyl linoleate	4	19 / 18 (12 pooled Int / 6 Ctrl)	mITT	34 (18-48) ‡ / 37 (23-47) ‡	61.5 / 33.3	NR	NR	FARS-Neuro: 66 (33-86) ‡ / 48 (38-66) ‡	NCT02445794
Marcotulli et al. 2016	Dose-escalation	Phase IIa	Interferon-γ 1b escalating (100, 150, 200 μg)	None (single arm)	5	9 / 9	NA	29.8 ± 6.0 / NA	66.7 / NA	NR	NR	SARA: 25.4 ± 10.2	EudraCT 2012-001881-14
Yiu et al. 2015	Non-randomized	NR	Resveratrol 5 g/day	Historical	12	27 / 24 (12 high-dose / NA)	NA	39.2 ± 7.7 / NA	25 / NA	19.4 ± 6.7 / NA	568 ± 212	FARS: 91.8 ± 26.0; SARA:	NCT01339884

Study	Design	Phase	Intervention (Dose)	Comparator	Duration (weeks)	Sample Size (N randomized / N analyzed)	Analysis Population	Age, years Mean ± SD (Int / Ctrl)	Female, % (Int / Ctrl)	Disease Duration, years Mean ± SD (Int / Ctrl)	GAA Repeats (shorter allele) Mean ± SD	Baseline Disease Severity Mean ± SD	Registration Number
												20.5 ± 7.9; ICARS: 49.1 ± 17.5	
Soragni et al. 2014	Crossover	Phase I	RG2833 (HDAC inhibitor) 30-240 mg	Placebo	0.14 (1 day)	20 / 20	Crossover	30.0 ± 8.1 / 30.0 ± 8.1	59.1 / 59.1	NR	1085 ± 785	NA (safety/PK study)	EudraCT 2011-000248-12
Arpa et al. 2013	Pilot study	NR	Darbepoetin alfa + Idebenone + Riboflavin	None (single arm)	32 (avg)	9 / 9	NA	28 ± 8 / NA	100 / NA	16.3§ / NA	NR	SARA: 20.7 ± 8.3	NR
Meier et al. 2012	OLE of RCT	NR	Idebenone 1350 or 2250 mg/day	Historical	52	68 / 68 (22 high / 46 low/placebo)	ITT	14.0 ± 2.72 / NA	54.5 / NA	NR	NR	FARS: 56.3 ± 12.3; SARA: 33.1 ± 9.7	NCT00697073
Abbruzzese et al. 2011	Single-arm pilot	Phase II	Deferiprone 30 mg/kg/day	None (single arm)	52	6 / 6	NA	36.5 ± 17.1 / NA	33.3 / NA	NR	NR	ICARS/UPDRS-III reported	NTC00907283
Lynch et al. 2010	RCT	Phase III	Idebenone 1350 or 2250 mg/day (high dose) †	Placebo	24	70 / 70 (24 high / 24 placebo)	ITT	13.4 ± 3.0 / 13.7 ± 2.8	58.3 / 66.7	5.3 ± 2.8 / 5.9 ± 3.9	725 ± 109 / 738 ± 130	FARS: 56.5 ± 11.6 / 55.9 ± 10.4; SARA: 36.0 ± 7.1 / 35.6 ± 7.0	NCT00537680
DiProspero et al. 2007	Dose-escalation	Phase I	Idebenone up to 75 mg/kg (1a); 60 mg/kg/day (1b)	None (single arm)	4 (Phase 1b)	93 / 14 (Phase 1b)	NA	23.2 ± 12.4 / NA	46.7 / NA	NR	NR	NA (safety/PK study)	NCT00015808
Boddaert et al. 2007	Open trial	Phase I/II	Deferiprone 20-30 mg/kg/day	Historical	24	9 / 9	NA	17.6 ± 3.5 / NA	77.8 / NA	7.8 ± 2.4 / NA	NR	ICARS: 34.4 ± 13.5	NCT00224640

Notes: *† Multi-arm trial; data shown for highest dose arm compared to placebo; ‡ Median (range) reported; § Disease duration from diagnosis; ¶ In vitro study, not a clinical trial* ***Abbreviations:*** *RCT, randomized controlled trial; Ext, extension; mITT, modified intent-to-treat; FAS, full analysis set; ITT, intent-to-treat; Int, intervention; Ctrl, control; NR, not reported; NA, not applicable; GAA, guanine-adenine-adenine triplet repeat; mFARS, modified Friedreich Ataxia Rating Scale; FARS, Friedreich Ataxia Rating Scale; SARA, Scale for Assessment and Rating of Ataxia; ICARS, International Cooperative Ataxia Rating Scale; TQT, thorough QT study; TID, three times daily; OLE, open-label extension; PK, pharmacokinetics; UPDRS, Unified Parkinson's Disease Rating Scale; NRF2, nuclear factor erythroid 2-related factor 2; HDAC, histone deacetylase; SD, standard deviation; wk, week; avg, average; prev, previously.*

Table S8. GRADE Evidence Quality Assessment for Primary and Secondary Outcomes.

Outcome	No. of Studies (Participants)	Study Design	Risk of Bias	Inconsistency	Indirectness	Imprecision	Publication Bias	Large Effect	Dose-Response	Confounding	Final Certainty Rating	Interpretation
**EFFICACY OUTCOMES:**												
mFARS change from baseline	3 (196: 99 Int / 97 Ctrl)	RCT	-1 (One study dose-ranging with small sample; some concerns in risk of bias assessment)	-1 (I² = 57.7%; moderate heterogeneity; contradictory directions; P-het = 0.10)	0 (Direct comparison in target population with validated measure)	-1 (CI crosses null: -3.35 to 0.94; n=196 vs ~400 needed; p=0.27)	0 (Cannot assess <10 studies; all registered)	0 (MD = -1.21, small effect)	0	0	⊕◯◯◯ Very Low	Very low confidence in effect estimate; true effect may be substantially different
FARS change from baseline	2 (140: 71 Int / 69 Ctrl)	RCT	-1 (Change score SD imputed using Cochrane method in Lynch 2010)	0 (I² = 8.8%; low heterogeneity; P-het >0.30)	0 (Direct measurement in target population)	-2 (Very wide CI: -2.33 to 1.95; small sample; clearly non-significant p=0.86)	0 (Cannot assess with 2 studies)	0 (MD = -0.19, negligible)	0	0	⊕◯◯◯ Very Low	Very low confidence; severely underpowered
SARA change from baseline	1 (48: 24 Int / 24 Ctrl)	RCT	-1 (SD imputed; otherwise low risk)	0 (NA - single study)	0 (Direct measurement)	-2 (Single small study; wide CI: -3.92 to 1.72; cannot pool)	0 (Cannot assess single study)	0	0	0	⊕◯◯◯ Very Low	Only one small study; very limited confidence
ICARS change from baseline	1 RCT (48: 24 Int / 24 Ctrl) + 1 single-arm	RCT + Single-arm	-1 (SD borrowed from Meier 2012; single-arm study high risk)	0 (NA - only 1 RCT)	0 (Direct assessment with validated scale)	-2 (Single small RCT; wide CI: -4.27 to 2.07; insufficient evidence)	0 (Cannot assess single RCT)	0	0	0	⊕◯◯◯ Very Low	Very limited evidence; high uncertainty
**SAFETY OUTCOMES:**												
Any adverse event	5 (351: 200 Int / 151 Ctrl)	RCT	0 (All RCTs had adequate blinding and AE ascertainment; low detection bias)	0 (I² = 0.0%; no heterogeneity; highly consistent; P-het >0.30)	0 (Direct assessment in relevant population)	0 (Narrow CI: 0.98 to 1.03; large sample n=351; precise estimate)	0 (Five studies; safety less subject to publication bias; no selective reporting)	0 (RR = 1.00, no effect)	0	0	⊕⊕⊕⊕ High	High confidence that true effect is close to estimated effect
Serious adverse events	4 (266: 153 Int / 113 Ctrl)	RCT	0 (Well-designed RCTs; clear SAE definitions; systematic monitoring)	-1 (I² = 36.0%; moderate heterogeneity; different SAE types)	0 (Direct measurement)	-2 (Very wide CI: 0.24 to 5.18; few events: 8 Int vs 5 Ctrl; underpowered for rare events)	0 (SAEs required reporting; low selective reporting risk)	0	0	0	⊕◯◯◯ Very Low	Wide CI indicates high uncertainty about true SAE risk

Outcome	No. of Studies (Participants)	Study Design	Risk of Bias	Inconsistency	Indirectness	Imprecision	Publication Bias	Large Effect	Dose-Response	Confounding	Final Certainty Rating	Interpretation
Withdrawal due to adverse events	4 (303: 176 Int / 127 Ctrl)	RCT	0 (Well-documented withdrawal reasons; low attrition bias)	0 (I^2 = 0.0%; no heterogeneity; consistent findings)	0 (Direct measurement of tolerability)	-2 (Wide CI: 0.40 to 4.19; few events: 9 Int vs 4 Ctrl; cannot rule out important difference)	0 (Systematically reported; low bias risk)	0	0	0	⊕⊕◯◯ Low	Limited confidence; true effect may differ substantially

GRADE Certainty Levels:* ⊕⊕⊕⊕ *High: *We are very confident that the true effect lies close to that of the estimate of the effect* ⊕⊕⊕◯ ***Moderate***: *We are moderately confident in the effect estimate; the true effect is likely to be close to the estimate, but there is a possibility that it is substantially different* ⊕⊕◯◯ ***Low***: *Our confidence in the effect estimate is limited; the true effect may be substantially different from the estimate* ⊕◯◯◯ ***Very Low***: *We have very little confidence in the effect estimate; the true effect is likely to be substantially different from the estimate.* ***Abbreviations:*** *mFARS, modified Friedreich Ataxia Rating Scale; FARS, Friedreich Ataxia Rating Scale; SARA, Scale for Assessment and Rating of Ataxia; ICARS, International Cooperative Ataxia Rating Scale; Int, intervention; Ctrl, control; RCT, randomized controlled trial; MD, mean difference; RR, risk ratio; CI, confidence interval; I^2, inconsistency index; SD, standard deviation; NA, not applicable; 0, no downgrade or upgrade; -1, downgrade one level; -2, downgrade two levels.*

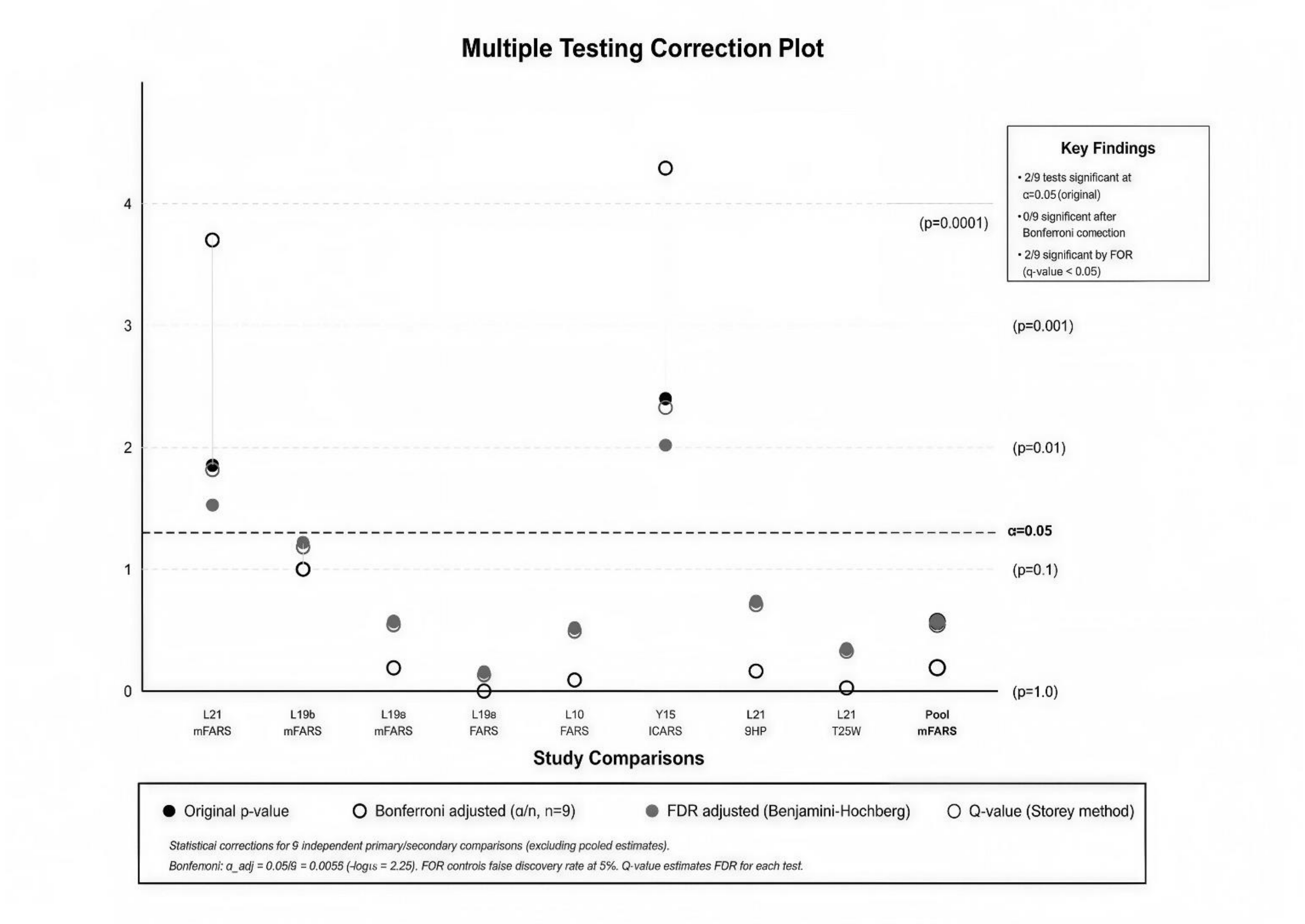

Figure S1. *(Multiple Testing Correction Plot).*

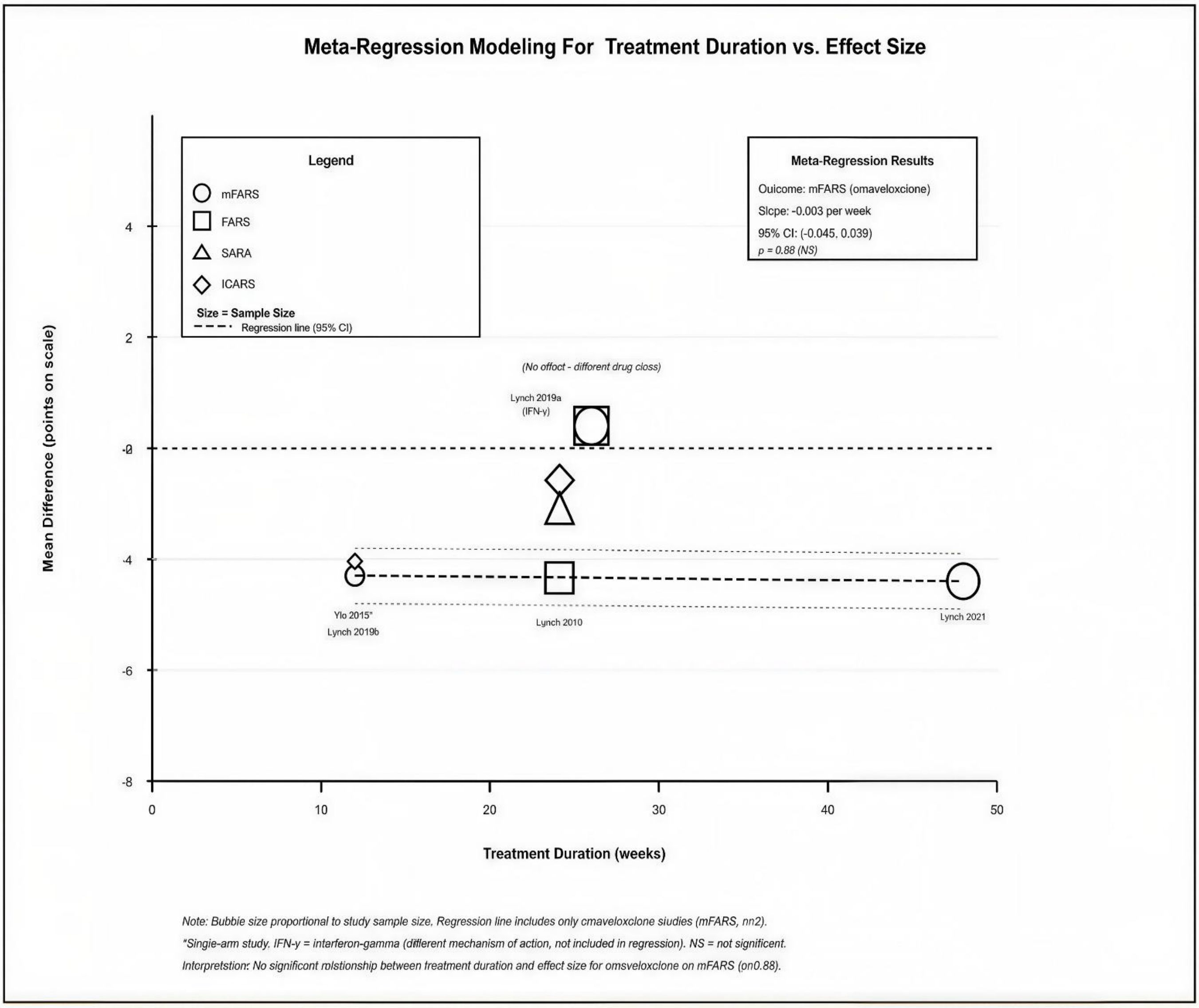

Figure S2: (*Meta-Regression Modeling For Treatment Duration vs Effect Size*).

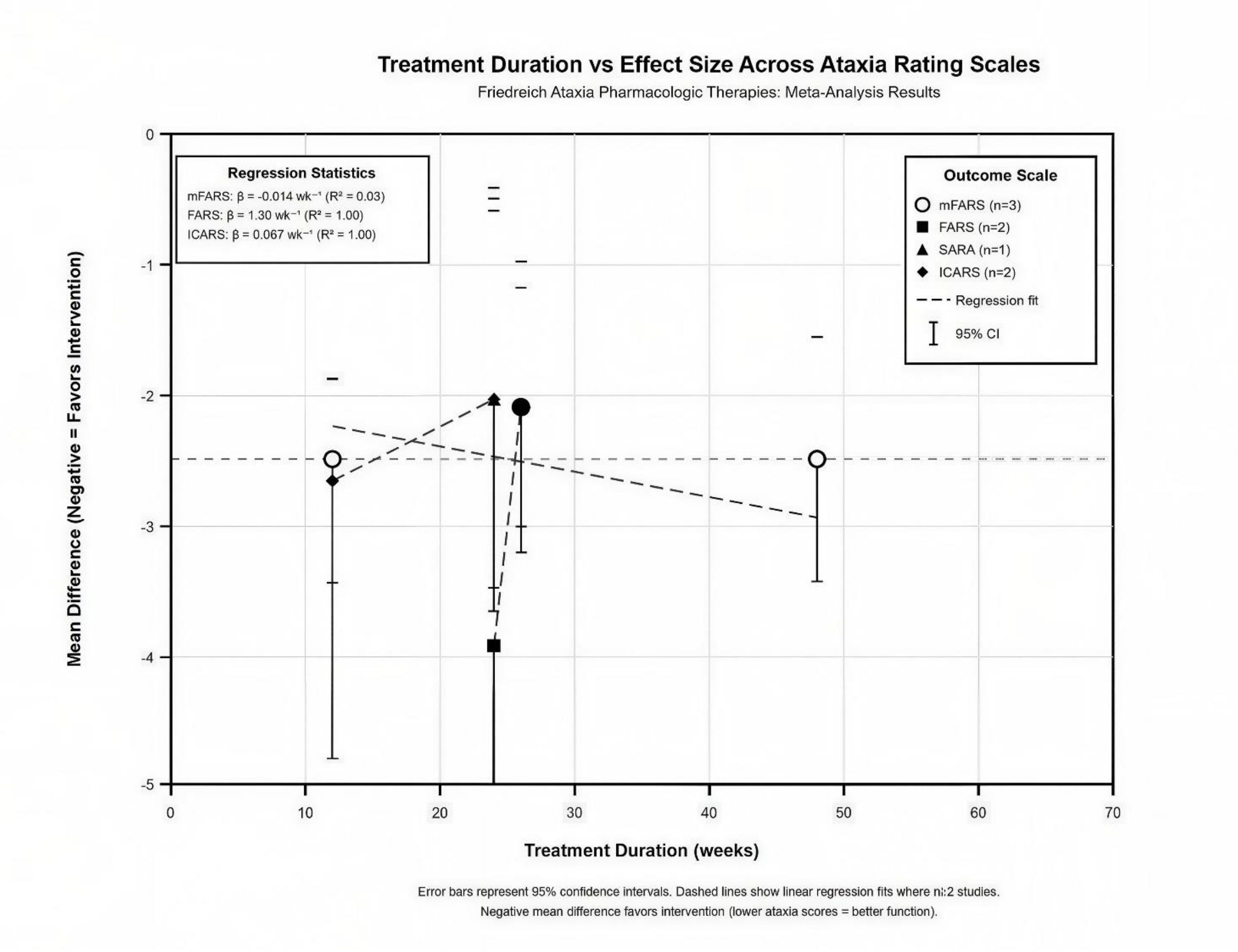

Figure S3: *Treatment Duration Vs Effect Size Across Ataxia Rating Scales*
